# Pennsylvania State Veterinary Medical Association

**Published:** 1901-04

**Authors:** 


### PENNSYLVANIA STATE VETERINARY MEDICAL ASSOCIATION.

THE annual meeting was opened at the Veterinary Department of the University of Pennsylvania on March 5, 1901. The meeting was called to order by President S. J. J. Harger at 10.30 A.M. The following members were present: John W. Adams, N. H. Allis, B. F. Bachman, W. G. Benner, U. S. G. Bieber, Charles W. Boyd, John L. Bradley, Francis Bridge, J. F. Butterfield, J. M. Carter, A. O. Cawley, M. J. Colllins, M. E. Conard, John M. Courtwright, H. P. Eves, H. B. Felton, J. T. Ferley, J. C. Foelker, W. H. Fry, Robert Gladfelter, Alexander Glass, Charles T. Goentner, C. R. Good, Elias A. Gruver, H. D. Hackler, S. J. J. Harger, Jacob Helmer, W. Horace Hoskins, J. D. Houldsworth, J. B. Irons, H. S. Jackson, George B. Jobson, Joseph Johnson, W. S. Kooker, S. D. Larzelere, Charles E. McGill, James R. Mahaffy, C. J. Marshall, D. Martin, J. C. McNeil, W. A. Meredith, J. C. Michener, P. M. Minster, M. Moriarty, E. S. Muir, Otto Noack, E. W. Newcomer, Leonard Pearson, J. R. Phillips, A. R. Potteiger, E. M. Ranck, G. B. Rayner, Thomas B. Rayner, James B. Rayner, John B. Raynor, M. Rectenwald, W. L. Rhoads, W. H. Ridge, James T. Ross, J. W. Sallade, Charles Schauffler, A. F. Schrieber, Samuel J. Swift, E. E. Terry, E. E. Tower, H. W. Turner, A. W. Wier, Charles Williams. In order to economize time the minutes of the previous meeting were not read.

President Harger gave the annual address, a paper entitled "The Synopsis of Veterinary Medicine." Among other things he said: "To-day convenes the twentieth annual meeting of this Association. This organization had its birth in 1882 in Donaldson Hall, Broad and Filbert Streets, Philadelphia. The meeting was called at the instigation of a Mr. Daniels, of Pittsburg, presumably in the interests of a sporting journal for which he solicited the professional and financial support of veterinarians. About thirty members from this city and various other parts of the State were present, and of this number about a dozen are on our roll at the present time. Dr. James McCoart, of this city, was the temporary chairman and Dr. James W. Sallade was the first president. Thus was, we might say, the accidental origin of our present association. Annual meetings, to which were subsequently added the semi-annual gatherings, were held interruptedly ever since. About six years ago, at the suggestion of the writer, the annual session was changed from one to two days. Yearly our list of membership has increased, and at present it numbers 145 out of a total of about 600 or 800 active practitioners in the State, the largest State organization in the United States. . . . .

"Of the 8000 veterinarians in the United States (9000 in North America) about 1500 are members of associations, and recruits from the ranks of the elder practitioners are constantly enlisted. To the educational influence disseminated by the national and State associations in permitting concentration of force, advancement in veterinary work, and increasing the curricula must be justly added certain legislative measures enacted by the several States.

"In Pennsylvania the first step in this direction was the Registration Act of 1889. Much as this law was criticised, many as its legal defects may have been, yet its operation was of undoubted value. It was not a complete panacea for illegal registration, but it had, besides, a moral effect upon the profession and paved the way for future remedies. In 1895 the State Board of Veterinary Examiners was authorized by the State preceded for a year or two by that of Ohio, which is the oldest. . . . .

"Few persons stop to recognize the vast diversified interests in this Commonwealth which the veterinarian must protect. The total value of the live-stock is over one hundred million of dollars. This includes 550,000 horses—a number exceeded only by New York and Illinois. Pennsylvania stands third in the United States in point of milk production—368,000,000 gallons in 1898, with a total yearly value of dairy products of over fifty millions of dollars. These animals require to be treated when they are sick; their wholesale destruction by the ravage of contagious diseases which, to the 58 per cent. of our population engaged in agriculture, means the loss of many fortunes, must be prevented; the causes and the remedies for obscure diseases, the comparative pathologist and therapeutist must discover; the live-stock must be improved by the introduction of male producers of the improved breeds, by judicious breeding and rational feeding; the animal food, for the safeguard of the citizen, should pass under the scrutinizing eye of the veterinarian. An educational movement which lies in our hands will help to accomplish our purpose. To this end the State as well as the public should give us aid, not only moral but material, to obtain such results as well as to educate the men of the future who must continue this work.

"It is with much regret that we learn of the closing of the Veterinary Department of Harvard University, and that the resources of this institution and of the New England States are insufficient to maintain this school intact."

The following officers were unanimously elected for the ensuing year: President, Dr. S. J. J. Harger; Vice-Presidents: First, W. L. Rhoads; Second, M. Moriarity; Third, Charles W. Boyd; Treasurer, Francis Bridge; Recording Secretary, C. J. Marshall; Corresponding Secretary, E. M. Ranck. Board of Trustees: Leonard Pearson, Thomas B. Rayner, W. Horace Hoskins, H. B. Felton, M. Rectenwald.

A recess of thirty minutes was ordered by the President for the purpose of paying dues and to give the Board of Censors time to transact their necessary business. After adjournment the Board of Censors reported as follows:

William W. Wilson, of Hartstown, Pa., was dropped from the list of members; Dr. Alexander Glass's case was satisfactorily adjusted; W. B. Miller, who is in arrears $11, was retained as a member. Several other were reported in arrears, but were held over for future consideration. E. G. Brittain's case was referred to the Board of Registration. Raymond Barber, of Newtown, Bucks County, who is pursuing a course in medicine, requested that his name be dropped from the society, which favor was granted.

The following new members were favorably reported and unanimously elected to membership: J. Morris Carter, of Philadelphia; Joseph Johnson, of Kirkwood; E. W. Newcomer, of Mount Joy; E. T. Cornman, of Philadelphia. Action was deferred in the case of P. A. Lehman, of York, because he was not present for an examination.

(To be continued.)

